# Age-Related Risk Factors and Complications of Patients With COVID-19: A Population-Based Retrospective Study

**DOI:** 10.3389/fmed.2021.757459

**Published:** 2022-01-11

**Authors:** Han Zhang, Yingying Wu, Yuqing He, Xingyuan Liu, Mingqian Liu, Yuhong Tang, Xiaohua Li, Guang Yang, Gang Liang, Shabei Xu, Minghuan Wang, Wei Wang

**Affiliations:** ^1^Department of Neurology, Tongji Medical College, Tongji Hospital, Huazhong University of Science and Technology, Wuhan, China; ^2^Department of Oncology, Tongji Medical College, Tongji Hospital, Huazhong University of Science and Technology, Wuhan, China; ^3^Wuhan Municipal Health Commission, Wuhan, China; ^4^Winning Health Technology Group Co., Ltd., Shanghai, China

**Keywords:** COVID-19, age, risk factors, complications, death

## Abstract

**Objective:** To study the differences in clinical characteristics, risk factors, and complications across age-groups among the inpatients with the coronavirus disease 2019 (COVID-19).

**Methods:** In this population-based retrospective study, we included all the positive hospitalized patients with COVID-19 at Wuhan City from December 29, 2019 to April 15, 2020, during the first pandemic wave. Multivariate logistic regression analyses were used to explore the risk factors for death from COVID-19. Canonical correlation analysis (CCA) was performed to study the associations between comorbidities and complications.

**Results:** There are 36,358 patients in the final cohort, of whom 2,492 (6.85%) died. Greater age (odds ration [*OR*] = 1.061 [95% *CI* 1.057–1.065], *p* < 0.001), male gender (*OR* = 1.726 [95% *CI* 1.582–1.885], *p* < 0.001), alcohol consumption (*OR* = 1.558 [95% *CI* 1.355–1.786], *p* < 0.001), smoking (*OR* = 1.326 [95% *CI* 1.055–1.652], *p* = 0.014), hypertension (*OR* = 1.175 [95% *CI* 1.067–1.293], *p* = 0.001), diabetes (*OR* = 1.258 [95% *CI* 1.118–1.413], *p* < 0.001), cancer (*OR* = 1.86 [95% *CI* 1.507–2.279], *p* < 0.001), chronic kidney disease (CKD) (*OR* = 1.745 [95% *CI* 1.427–2.12], *p* < 0.001), and intracerebral hemorrhage (ICH) (*OR* = 1.96 [95% *CI* 1.323–2.846], *p* = 0.001) were independent risk factors for death from COVID-19. Patients aged 40–80 years make up the majority of the whole patients, and them had similar risk factors with the whole patients. For patients aged <40 years, only cancer (*OR* = 17.112 [95% *CI* 6.264–39.73], *p* < 0.001) and ICH (*OR* = 31.538 [95% *CI* 5.213–158.787], *p* < 0.001) were significantly associated with higher odds of death. For patients aged >80 years, only age (*OR* = 1.033 [95% *CI* 1.008–1.059], *p* = 0.01) and male gender (*OR* = 1.585 [95% *CI* 1.301–1.933], *p* < 0.001) were associated with higher odds of death. The incidence of most complications increases with age, but arrhythmias, gastrointestinal bleeding, and sepsis were more common in younger deceased patients with COVID-19, with only arrhythmia reaching statistical difference (*p* = 0.039). We found a relatively poor correlation between preexisting risk factors and complications.

**Conclusions:** Coronavirus disease 2019 are disproportionally affected by age for its clinical manifestations, risk factors, complications, and outcomes. Prior complications have little effect on the incidence of extrapulmonary complications.

## Introduction

The coronavirus disease 2019 (COVID-19) is a novel coronavirus infection disease causing respiratory failure and a high death rate in vulnerable populations (1). Despite increasing clinical experience and a rapidly expanding of literature on diverse aspects of COVID-19, the relationship between common risk factors and mortality from COVID-19 remains incompletely understood.

Prior studies have identified greater age, male sex, and metabolic comorbidities, such as hypertension and diabetes as risk factors for poor outcomes in patients with COVID-19 (2, 3). These comorbidities are more common among older age groups, and overwhelming evidence from around the world suggests that age itself is the most significant risk factor for severe disease and death from COVID-19 (3). Emerging data suggest that the effect of comorbidities on COVID-19 may be age-dependent (4–6). However, due to the small sample size of previous studies, it is challenging to isolate the independent effects of these clustered characteristics as a function of patient age. Besides, COVID-19 can affect multiple organ systems (7, 8), and cause complications, such as acute respiratory distress syndrome (ARDS), respiratory failure, acute myocardial infarction (AMI), acute kidney injury (AKI), coagulopathy (9), sepsis, or systemic inflammation (10). Since these complications are extremely life-threatening, their prevalence can act as determining factors in the morbidity and mortality rates of the disease (11, 12). However, there is a lack of research about the effects of age on the incidence of complications and the association between complications and prior comorbidities of patients (13).

To address these deficiencies in the literature, in this study we determine clinical characteristics, risk factors for death, and complications across age groups in the whole dataset of inpatients with COVID-19 across Wuhan city, China. Wuhan has emerged as the original epicenter of COVID-19, with some 50,000 individuals infected and nearly 4,000 deaths recorded during the early phase of this pandemic. Using this unique cohort, we explored the association between complications and prior comorbidities of patients.

## Methods

### Study Design and Participants

This is a retrospective cohort study using data collected from all inpatients in Wuhan with positive COVID-19 test results recorded from December 29, 2019, when the patients were first admitted to hospital, and extending to April 15, 2020, this being the first day with no new cases declared in Wuhan city. The compilation of data was coordinated by the Wuhan Health Commission, which mandated the reporting of clinical information from every designated hospital (such as, 61 Wuhan hospitals and mobile cabin hospitals) in Wuhan that had admitted patients with laboratory-confirmed COVID-19. We included all the inpatients with positive COVID-19 in this administrative dataset. We excluded patient records with incomplete clinic data. The follow-up of patients continued through July 1, 2020. This study was designed by the authors and approved with the provision of a waiver of authorization and informed consent by the ethics committee of Tongji Hospital (IRB ID: TJ-C20200121). The data are anonymous, and the requirement for informed consent was therefore waived.

### Data Collection and Data Extraction

Demographic, clinical, laboratory, treatment, and outcome data were extracted from electronic medical records using a standardized data collection and processing method. Detailed information about procedures is provided in Supplementary Materials. The laboratory data were collected at the time of admission of the patient.

### Definitions

Age groups were set as <18, 18–40, 40–60, 60–80, and >80 years since differences in mortality were reported according to a previous study (14). The clinical outcomes were defined as hospital discharge or death. COVID-19 related deaths were defined as those occurring in patients who tested positive for severe acute respiratory syndrome coronavirus 2 (SARS-CoV-2) through reverse transcription-PCR (RT-PCR). The cardiac injury was defined as an elevation of at least one of the three cardiac biomarkers above the 99th percentile upper reference limit, or if new abnormalities were shown in electrocardiography and echocardiography (15). Acute respiratory distress syndrome (ARDS) was defined according to the Berlin definition (16). Respiratory failure was defined as the requirement for oxygen supplementation or mechanical ventilation (17). AKI was identified according to the Kidney Disease: Improving Global Outcomes definition (18). Cardiac arrhythmias were recorded according to the ECG measured upon initial admission and duration of the hospital stay, or were collected from the medical records of patients (19). Coagulopathy was defined as a 3-s prolongation of prothrombin time or a 5-s prolongation of activated partial thromboplastin time, or by any evidence of pulmonary embolism, lower limb venous thrombosis, arterial embolism, or disseminated intravascular coagulation (DIC) (20). Acute liver injury (ALI) was defined as a state in which the blood laboratory results of patients met at least one of three criteria: serum total bilirubin of 1.0 mg/dl or greater; aspartate aminotransferase of 41 IU/L or greater; and alanine aminotransferase of 41 IU/L or greater. AMI and acute ischemic stroke (AIS) was diagnosed as previously described (21, 22). Gastrointestinal bleeding was defined as the presence of hematemesis, melena, or hematochezia. Sepsis was defined according to The Third International Consensus Definitions for Sepsis and Septic Shock (Sepsis-3) (23).

### Statistical Analysis

Continuous variables with non-normal distributions were described as a median interquartile range (IQR). Categorical variables were described as number and percentage (%). Comparison between two groups was performed with Wilcoxon test for non-parametric variables, Fisher's exact test or the chi-square test was performed for categorical variables. Multivariate logistic regression models were developed to explore the relative contributions of risk factors. Variables with *P* < 0.1 in univariate regression were included in multiple regression. There were no significant interactions between variables enrolled in multivariate analyses. The value of *P* < 0.05 was considered statistically significant. Canonical correlation analysis (CCA) was used to analyze the correlation between preexisting risk factors and the incidence of complications. The sign of the standardized coefficient indicates the direction in which the raw variable influences the canonical variate. The magnitude of the standardized coefficient represents the magnitude of the influence of variable on the canonical variate. Data preprocessing and statistical analysis were calculated with Ubuntu 16.04.6 and an R-studio server (R-4.0.3 R Foundation for Statistical Computing). CCA was performed using the “matcor” function in the CCA package (detail in Supplementary Materials).

### Data Availability

The data that support the findings of this study were available from the corresponding author upon reasonable request.

## Results

### Baseline Characteristics and Outcomes of Patients With COVID-19 in Different Age Groups

**Figure 1:**
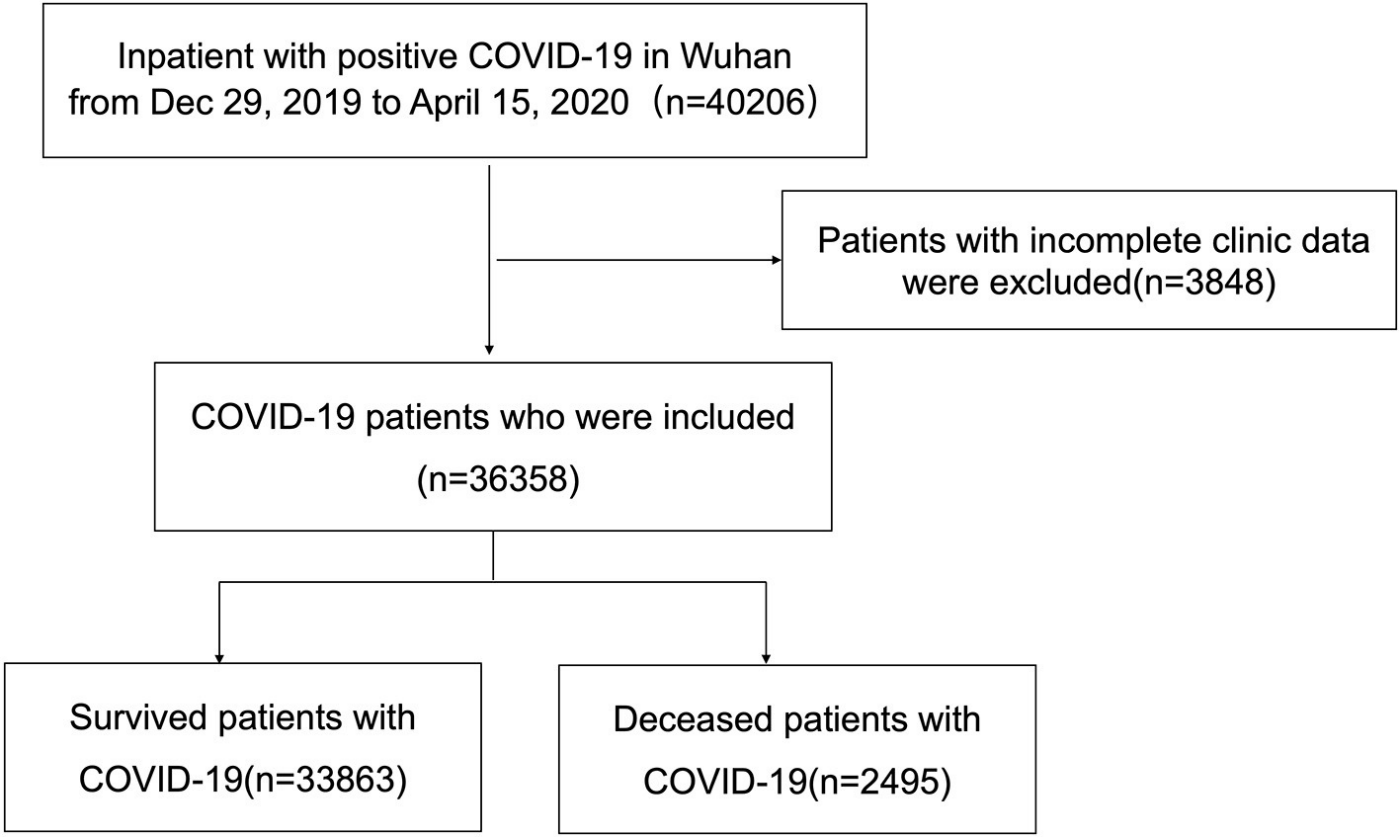
Flowchart of patient inclusion and exclusion.

In total, 40,206 patients were found positive for COVID-19 and received in-hospital treatment in Wuhan, Hubei Province. After excluding patients with critical missing data, the final cohort consisted of 36,358 patients (Figure 1). There were 473, 6,106, 12,950, 14,805, and 2,497 patients with COVID-19 included in the age group of <18, 18–40, 40–60, 60–80, and >80 years old, respectively. The most common symptoms on admission were fever (66.1%) and cough (62.0%), followed by shortness of breath (39.7%) and fatigue (38.5%). Patients aged <18 years had a lower incidence in almost all the symptoms but the running nose. Patients aged between 40 and 80 years had a higher incidence of fever, cough, muscle soreness, and fatigue. Hemoptysis and anorexia showed increasing incidence with age. Patients aged older than 80 years showed a significantly increased incidence of disturbance of consciousness (4.5%).

Patients aged <18 years had less comorbidities. Patients aged >40 years had a greater prevalence of smoking and alcohol consumption. The incidence of comorbidities, such as hypertension, diabetes mellitus, hyperlipidemia, heart disease, tuberculosis, malignant tumor, kidney disease, chronic obstructive pulmonary disease (COPD), cerebral hemorrhage, and ischemic stroke increased with age groups. Patients aged >80 years had a significantly greater history of heart disease. Patients aged <60 years were more likely to have a history of liver disease.

Elderly patients showed a greater prevalence of positive laboratory tests, such as leukocytosis, lymphopenia, decreased platelet count, hemoglobinemia, lower albumin, increased D-D-dimer, and prolonged prothrombin times. ALT elevation occurred more frequently in younger patients. The mortality rate of patients with COVID-19 increased with age, with patients aged over 80 years having a particularly high mortality rate (21.6% vs. 0.8%, 1.0%, 3.4%, 9.8%) (Table 1).

**Table 1:** Baseline characteristics and outcomes of patients with coronavirus disease 2019 (COVID-19) in different age groups.

**characteristic**	**All**	**≤18**	**>18, ≤40**	**>40, ≤60**	**>60, ≤80**	**>80**	***P***
*n*	36,358	473	5,633	12,950	14,805	2,497	
Age	59[47.0, 69.0]	9.00 [4.00, 14.00]	34.00[30.00, 37.00]	52[48.0, 57.0]	68[64.0, 72.0]	85[83.0,88.0]	
Sex(female/male)	18,696/1766 (51.4/48.6)	208/265 (44.0/56.0)	2,760/2,873 (49.0/51.0)	6,855/6,095 (52.9/47.1)	7,604/7,201 (51.4/48.6)	1,269/1,228 (50.8/49.2)	<0.001
Systolic pressure	126.0 [114.0, 138.0]	114.50 [100.00, 122.75]	120.00 [110.00, 130.00]	125.0 [113.0, 135.0]	130.0 [116.0, 141.0]	130.0 [118.0, 144.0]	<0.001
Diastolic pressure	80.0 [72.0, 90.0]	71.50 [61.00, 80.00]	80.00 [71.00, 88.00]	80.0 [74.0, 92.0]	80.0 [73.0, 90.0]	78.0 [70.0, 87.0]	<0.001
Smoking	794 (2.2%)	4 (0.8)	304 (5.4)	258 (2.0)	1,407 (2.7)	45 (1.8)	<0.001
Drinking	2,697 (7.4)	0 (0.0)	84 (1.5)	1,024 (7.9)	1,193 (8.1)	172 (6.9)	<0.001
Diabetes	3,931 (10.8)	3 (0.6)	170 (3.0)	1,101 (8.5)	2,267 (15.3)	390 (15.6)	<0.001
Hypertension	8,498 (23.4)	0 (0.0)	187 (3.3)	2,205 (17.0)	4,993 (33.7)	1,113 (44.6)	<0.001
Hyperlipidemia	459 (1.3)	0 (0.0)	22 (0.4)	153 (1.2)	250 (1.7)	34 (1.4)	<0.001
Heart disease	2,452 (6.7)	3 (0.6)	75 (1.3)	359 (2.8)	1,446 (9.6)	571 (20.1)	<0.001
Cancer	824 (2.3)	6 (1.3)	41 (0.7)	257 (2.0)	427 (2.9)	93 (3.7)	<0.001
COPD	850 (2.3)	0 (0.0)	14 (0.2)	125 (1.0)	495 (3.3)	216 (8.7)	<0.001
Tuberculosis	506 (1.4)	1 (0.2)	57 (1.0)	157 (1.2)	229 (1.5)	62 (2.5)	<0.001
Chronic kidney disease	924 (2.5)	1 (0.2)	78 (1.4)	315 (2.4)	421 (2.8)	109 (4.4)	<0.001
Liver disease	1,189 (3.3)	4 (0.8)	186 (3.3)	511 (3.9)	413 (2.8)	75 (3.0)	<0.001
Intracerebral hemorrhage	178 (0.5)	0 (0.0)	8 (0.1)	42 (0.3)	187 (0.6)	41 (1.6)	<0.001
Asthma	386 (1.1)	3 (0.6)	52 (0.9)	134 (1.0)	165 (1.1)	32 (1.3)	0.48
History of stroke	1,160 (3.2)	0 (0.0)	8 (0.1)	136 (1.1)	668 (4.5)	348 (13.9)	<0.001
Fever	24,026 (66.1)	266 (56.2)	3,725 (66.1)	8,799 (67.9)	9,831 (66.4)	1,405 (56.3)	<0.001
Cough	22,525 (62.0)	209 (44.2)	3,345 (59.4)	8,164 (63.0)	9,345 (63.1)	1,462 (58.6)	<0.001
Hemoptysis	1,504 (4.1)	7 (1.5)	200 (3.6)	480 (3.7)	679 (4.6)	138 (5.5)	<0.001
Muscle soreness	4,006 (11.0)	11 (2.3)	563 (10.0)	1,457 (11.3)	1,737 (11.7)	238 (9.5)	<0.001
Fatigue	13,986 (38.5)	46 (9.7)	1,857 (33.0)	4,909 (37.9)	6,230 (42.1)	944 (37.8)	<0.001
Running nose	845 (2.3)	30 (6.3)	128 (2.3)	272 (2.1)	357 (2.4)	58 (2.3)	<0.001
Pharyngalgia	2,537 (7.0)	13 (2.7)	417 (7.4)	903 (7.0)	1,034 (7.0)	170 (6.8)	0.005
Shortness of breath	14,445 (39.7)	25 (5.3)	1,699 (30.2)	5,084 (39.3)	6,622 (44.7)	1,015 (40.6)	<0.001
Chest pain	2,328 (6.4)	9 (1.9)	345 (6.1)	810 (6.3)	999 (6.7)	165 (6.6)	<0.001
Anorexia	4,882 (13.4)	11 (2.3)	582 (10.3)	1,567 (12.1)	2,260 (15.3)	462 (18.5)	<0.001
Nausea and vomiting	3,821 (10.5)	39 (8.2)	604 (10.7)	1,283 (9.9)	1,614 (10.9)	281 (11.3)	0.019
Diarrhea	4,762 (13.1)	24 (5.1)	770 (13.7)	1,675 (12.9)	1,993 (13.5)	300 (12.0)	<0.001
Stomachache	1,988 (5.5)	15 (3.2)	311 (5.5)	621 (4.8)	860 (5.8)	81 (7.2)	<0.001
Deadache	2,773 (7.6)	17 (3.6)	458 (8.1)	1,026 (7.9)	1,091 (7.4)	181 (7.2)	0.002
Dizziness	2,093 (5.8)	6 (1.3)	343 (6.1)	717 (5.5)	854 (5.8)	173 (6.9)	<0.001
Disturbance of consciousness	404 (1.1)	2 (0.4)	26 (0.5)	77 (0.6)	187 (1.3)	112 (4.5)	<0.001
BWC, >10 ×10^9^ cells/L	1,858 (7.37)	5 (3.5)	180 (4.7)	478 (5.4)	975 (9.2)	220 (12.1)	<0.001
LC, <0.8 ×10^9^ cells/L	6,354 (20.84)	6 (3.8)	419 (9.3)	1,863 (17.0)	3,313 (25.9)	753 (36.3)	<0.001
Platelets, <100 ×10^9^ cells/L	1,284 (4.39)	3(1.9)	81 (1.8)	364 (3.3)	655 (5.1)	181 (8.7)	<0.001
Hemoglobin, <90 g/L	1,196 (3.93)	5 (3.2)	69 (1.5)	342 (3.1)	572 (4.5)	208 (10.0)	<0.001
Albumin, <35g/L	8,005 (29.01)	1 (0.7)	382 (9.3)	2,015 (20.4)	4,585 (39.7)	1,022 (53.6)	<0.001
ALT, >40 u/L	6,386 (23.05)	20 (13.2)	1,156 (28.2)	2,480 (25.0)	2,480 (21.4)	250 (13.0)	<0.001
Prothrombi*n* time, >16s	1,769 (7.03)	15 (11.3)	233 (6.4)	536 (6.1)	774 (7.2)	211 (11.6)	<0.001
D-D dimer, >0.5 μg/L	13,211 (36.34)	29 (23.1)	1,083 (28.6)	3,567 (38.01)	6,935 (60.85)	1,597 (82.83)	<0.001
Die	2,492 (6.9)	4 (0.8)	64 (1.1)	435 (3.4)	1,452 (9.8)	539 (21.6)	<0.001

*COPD, chronic obstructive pulmonary disease; COVID-19, coronavirus disease 2019; BWC, blood white cells; LC, lymphocyte; ALT, Alanine aminotransferase. Data are reported as a number of patients and percentage in the age group (%) for categorical variables and median (interquartile range [IQR]) for continuous variables*.

### Analysis of Risk Factors for Death in Patients With COVID-19

**Figure 2:**
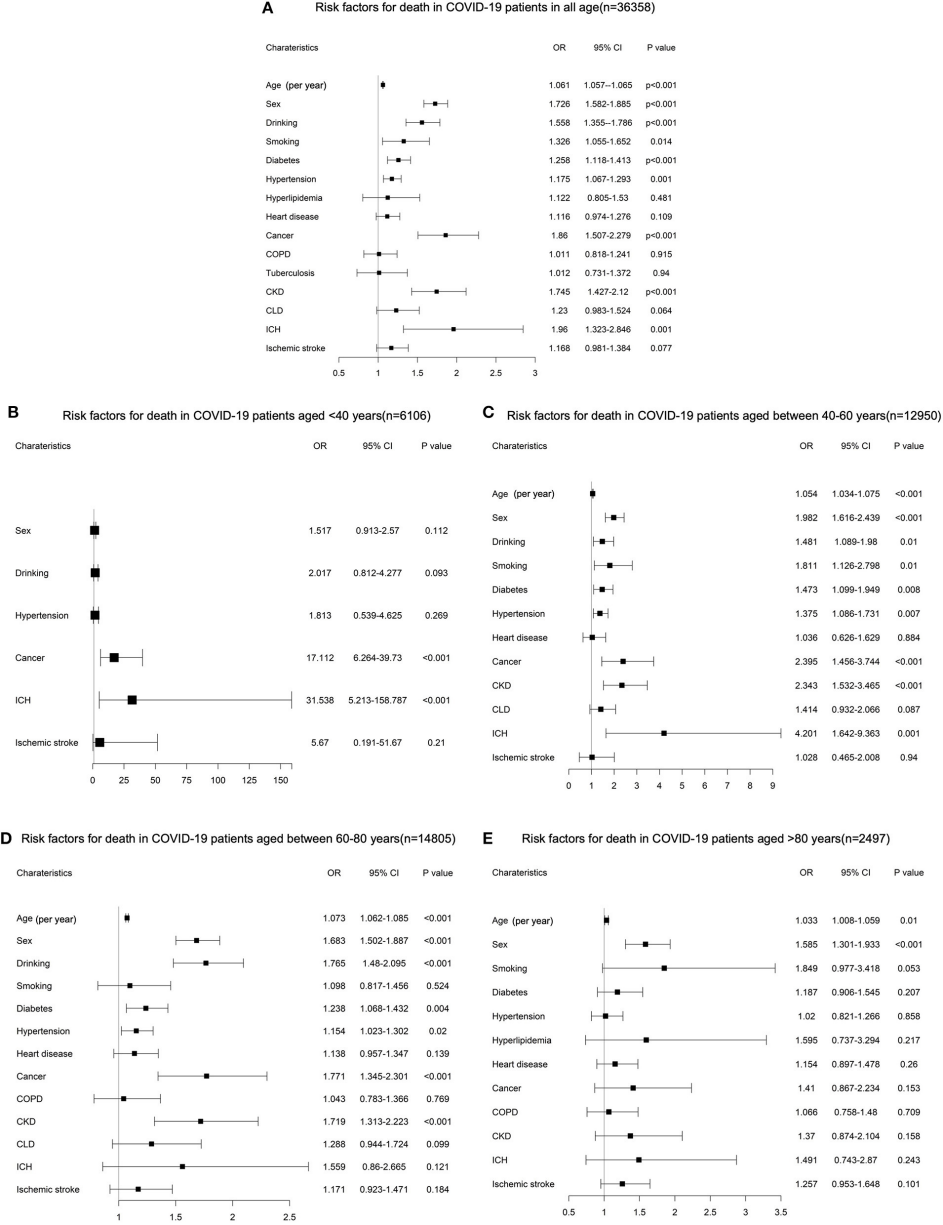
**(A)** Multivariable logistic regression analysis for risk factors of death in the entire cohort of patients with coronavirus disease 2019 (COVID-19). **(B)** Multivariable logistic regression analysis for risk factors of death in COVID-19 patients aged <40 years. **(C)** Multivariable logistic regression analysis for risk factors of death in COVID-19 patients aged between 40 and 60 years. **(D)** Multivariable logistic regression analysis for risk factors of death COVID-19 patients aged between 60 and 80 years. **(E)** Multivariable logistic regression analysis for risk factors of death COVID-19 patients aged >80 years.

To investigate the risk factors for death in patients with COVID-19, we performed a bilateral logistic regression analysis. After adjusting for age, sex, and comorbidities, multivariable logistic regression analysis showed that greater age (*OR* = 1.061 [95% *CI* 1.057–1.065], *p* < 0.001), male gender (*OR* = 1.726 [95% *CI* 1.582–1.885], *p* < 0.001)], alcohol consumption (*OR* = 1.558 [95% *CI* 1.355–1.786], *p* < 0.001), smoking (*OR* = 1.326 [95% *CI* 1.055–1.652], *p* = 0.014), hypertension (*OR* = 1.175 [95% *CI* 1.067–1.293], *p* = 0.001), diabetes (*OR* = 1.258 [95% *CI* 1.118–1.413], *p* < 0.001), cancer (*OR* = 1.86 [95% *CI* 1.507–2.279], *p* < 0.001), chronic kidney disease (CKD) (*OR* = 1.745 [95% *CI* 1.427–2.12], *p* < 0.001), and intracerebral hemorrhage (ICH) (*OR* = 1.96 [95% *CI* 1.323–2.846], *p* = 0.001) were independent risk factors for death of COVID-19 (Figure 2A).

The differences among age groups were found when results were stratified by age groups. For patients aged <40 years, only cancer (*OR* = 17.112 [95% *CI* 6.264–39.73], *p* < 0.001) and ICH (*OR* = 31.538 [95% *CI* 5.213–158.787], *p* < 0.001) were associated with higher odds of death (Figure 2B). For people aged 40–60 years, risk factors are almost the same for all patients, but cancer (*OR* = 2.395 [95% *CI* 1.456–3.744], *p* < 0.001), CKD (*OR* = 2.343 [95% *CI* 1.532–3.465], *p* < 0.001), and ICH (*OR* = 4.201 [95% *CI* 1.642–9.363], *p* = 0.001) had an obvious higher *OR* for death (Figure 2C). For people aged 60–80 years, older age (*OR* = 1.073 [95% *CI* 1.062–1.085], *p* < 0.001), male gender (*OR* = 1.683 [95% *CI* 1.502–1.887], *p* < 0.001), alcohol consumption (*OR* = 1.765 [95% *CI* 1.48–2.095], *p* < 0.001), diabetes (*OR* = 1.238 [95% *CI* 1.068–1.432], *p* = 0.004), hypertension (*OR* = 1.154 [95% *CI* 1.023–1.302], *p* = 0.02), cancer (*OR* = 1.771 [95% *CI* 1.345–2.301], *p* < 0.001), and CKD (*OR* = 1.719 [95% *CI* 1.313–2.223], *p* < 0.001) were associated with higher odds of death (Figure 2D). For people aged >80 years, only age (*OR* = 1.033 [95% *CI* 1.008–1.059], *p* = 0.01) and male gender (*OR* = 1.585 [95% *CI* 1.301–1.933], *p* < 0.001) were associated with higher odds of death (Figure 2E).

### Baseline Characteristics of Deceased Patients With COVID-19 in Different Age Groups

There were 2,495 (6.85%) patients who died during their hospitalization. Among them, men were over-represented across all age groups. Patients aged <40 (10.6 and 1.5%) and >80 (7.1 and 3.3%) years were less likely to smoke and drink. The proportion of patients with diabetes, hypertension, hyperlipidemia, COPD, tuberculosis, CKD, prior stroke increased with age groups, but patients in the 40–60 years group had higher rates of CKD and CLD. Patients in <40 age group had significant higher rates of ICH (4.5% vs. 1.6%, 1.1%, 2.6%), and cancer (9.1% vs. 4.8%, 4.9%, 5.0%) although without statistically significant.

Fever (62.1 and 64% vs. 79.5 and 75.4%), cough (56.1 and 61.8% vs. 71.0 and 65.6%), and shortness of breath (48.5 and 53.2% vs. 60.5 and 60.7%) were less common in patients aged <40 years and > 80 years, and patients aged >80 years showed a significantly increased incidence of disturbance of consciousness (11.3 vs. 6.1%, 5.5, 6.7%).

Regarding laboratory test results, leukocytosis was more common in deceased patients with COVID-19 in the group aged 60–80 years old. Lymphopenia, eosinophil, and basophilopenia were seen most frequently in the group aged 40–80 years. A significantly higher proportion of decease patients younger than 40 years of age had reduced hemoglobin (17 vs. 11.0%, 10.9, 11.0%). Reduced albumin was more common in deceased patients aged >60 (68.9 and 67.8% vs. 38.3 and 53.1%). The proportion of cases with increased D-D dimer rose with increasing age groups of deceased patients with COVID-19 (Table 2).

**Table 2:** Baseline characteristics and outcomes of deceased patients with COVID-19 in different age groups.

**characteristic**	**Overall**	**≤40**	**>40, ≤60**	**>60, ≤80**	**>80**	***p***
*n*	2,492	66	435	1,452	539	
Age	71.0 [63.0, 79.0]	34.0 [30.0, 38.0]	55.0 [50.0, 57.0]	70.0 [66.0, 75.0]	85.0 [83.0, 89.0]	<0.001
Sex (female/male)	958/1,534 (38.4/61.6)	25/41 (37.9/62.1)	153/282 (35.2/64.8)	558/894 (38.4/61.6)	222/317 (41.2/58.8)	0.297
Systolic pressure	125 [105.0, 140.0]	120 [104.2,132.2]	122 [100.5,135.0]	125.5 [103.0, 140.0]	128 [112.5, 141.0]	0.003
diastolic pressure	80.0 [70.2, 93.0]	83.0 [78.0, 93.2]	81.0 [74.0, 98.0]	80.0 [71.0, 94.0]	78.00 [70.0, 87.0]	<0.001
Smoking	295 (11.8)	7 (10.6)	59 (13.6)	191 (13.2)	38 (7.1)	0.001
Drinking	109 (4.4)	1 (1.5)	25 (5.7)	65 (4.5)	18 (3.3)	0.198
Diabetes	458 (18.4)	1 (1.5)	68 (15.6)	289 (19.9)	100 (18.6)	0.001
Hypertension	987 (39.6)	5 (7.6)	124 (28.5)	597 (41.1)	261 (48.4)	<0.001
Hyperlipidemia	48 (1.9)	0 (0.0)	8 (1.8)	28 (1.9)	12 (2.2)	0.666
Heart disease	355 (14.2)	2 (3.0)	21 (4.8)	197 (13.6)	135 (25.0)	<0.001
Cancer	125 (5.0)	6 (9.1)	21 (4.8)	71 (4.9)	27 (5.0)	0.497
COPD	126 (5.1)	0 (0.0)	5 (1.1)	63 (4.3)	58 (10.8)	<0.001
Tuberculosis	49 (2.0)	0 (0.0)	8 (1.8)	24 (1.7)	17 (3.2)	0.109
Chronic kidney disease	142 (5.7)	2 (3.0)	30 (6.9)	77 (5.3)	33 (6.1)	0.451
Liver disease	103 (4.1)	2 (3.0)	29 (6.7)	54 (3.7)	18 (3.3)	0.033
Intracerebral hemorrhage	40 (1.6)	3 (4.5)	7 (1.6)	16 (1.1)	14 (2.6)	0.026
Asthma	34 (1.4)	1 (1.5)	6 (1.4)	21 (1.4)	6 (1.1)	0.953
History of stroke	202 (8.1)	1 (1.5)	9 (2.1)	94 (6.5)	98 (18.2)	<0.001
Faver	1,827 (73.3)	41 (62.1)	346 (79.5)	1,095 (75.4)	345 (64.0)	<0.001
Cough	1,632 (65.5)	37 (56.1)	309 (71.0)	953 (65.6)	333 (61.8)	0.008
Hemoptysis	118 (4.7)	2 (3.0)	21 (4.8)	70 (4.8)	25 (4.6)	0.926
Muscle soreness	252 (10.1)	10 (15.2)	52 (12.0)	142 (9.8)	48 (8.9)	0.212
Fatigue	1,049 (42.1)	20 (30.3)	179 (41.1)	626 (43.1)	224 (41.6)	0.203
Running nose	66 (2.6)	1 (1.5)	10 (2.3)	34 (2.3)	21 (3.9)	0.229
Pharyngalgia	170 (6.8)	3 (4.5)	32 (7.4)	102 (7.0)	33 (6.1)	0.743
Shortness of breath	1,463 (58.7)	32 (48.5)	263 (60.5)	881 (60.7)	287 (53.2)	0.006
Chest pain	169 (6.8)	4 (6.1)	36 (8.3)	98 (6.7)	31 (5.8)	0.476
Anorexia	457 (18.3)	9 (13.6)	72 (16.6)	264 (18.2)	112 (20.8)	0.254
Nausea and vomiting	292 (11.7)	14 (21.2)	50 (11.5)	163 (11.2)	65 (12.1)	0.104
Diarrhea	369 (14.8)	7 (10.6)	72 (16.6)	218 (15.0)	72 (13.4)	0.404
Stomachache	155 (6.2)	5 (7.6)	24 (5.5)	84 (5.8)	42 (7.8)	0.343
Deadache	197 (7.9)	1 (1.5)	36 (8.3)	121 (8.3)	39 (7.2)	0.214
Dizziness	184 (7.4)	4 (6.1)	25 (5.7)	101 (7.0)	54 (10.0)	0.052
Disturbance of consciousness	187 (7.5)	4 (6.1)	24 (5.5)	98 (6.7)	61 (11.3)	0.002
BWCs, >10 ×10^9^/L	541 (31.3)	8(17.4)	79 (26.0)	363 (35.2)	91 (26.2)	<0.001
LCs, <0.8 ×10^9^/L	1,209 (61.4)	23 (43.4)	200 (56.5)	758 (64.7)	228 (58.3)	0.002
Platelets, <100 ×10^9^/L	340 (17.3)	10 (18.9)	47 (13.2)	207 (17.7)	76 (19.5)	0.132
Hemoglobin, <90 g/L	219 (11.1)	9 (17.0)	39 (11.0)	128 (10.9)	43 (11.0)	<0.001
Albumin, <35 g/L	1,201 (65.1)	18 (38.3)	173 (53.1)	753 (68.9)	257 (67.8)	<0.001
ALT, >40 u/L	537 (29.1)	15 (31.9)	101 (31.0)	344 (31.4)	77 (20.3)	<0.001
Prothrombin time, >16s	303 (18.4)	9 (18.8)	47 (16.3)	184 (18.9)	63 (18.6)	0.803
D-D dimer, >0.5μg/L	1,477(81.6)	27(51.92)	229(70.9)	910(84.34)	311(87.36)	<0.001

*COPD, chronic obstructive pulmonary disease; COVID-19, coronavirus disease 2019; BWC, blood white cells; LC, lymphocyte; ALT, alanine aminotransferase. Data are reported as number of patients and percentage in the age group (%) for categorical variables and median (IQR) for continuous variables*.

### Age-Related Complications in Deceased Patients With COVID-19

Patients with COVID-19 are apt to die from multiple complications. The comparison between complications in deceased patients according to age groups is shown in Table 3. The most common complications included ARDS (41.3%), respiratory failure (71.4%), and extrapulmonary complications, such as acute myocardial infarction (2.1%), acute myocardial injury (15.3%), arrhythmia (5.2%), acute kidney injury (19.4%), acute liver injury (19.0%), gastrointestinal bleeding (2.0%), acute ischemic stroke (1.5%), sepsis (10.9%), and coagulopathy (22.5%). The incidence of most complications increased with increasing age, with respiratory failure (*p* = 0.005), acute myocardial infarction (*p* = 0.049), acute myocardial injury (*p* = 0.001), AKI (*p* = 0.014), acute liver injury (*p* = 0.039), and acute ischemic stroke (*p* = 0.018) attaining statistical significance. However, arrhythmias (7.6% vs. 2.5, 5.6, 6.1%), gastrointestinal bleeding (4.5% vs. 2.3, 1.9, 1.5%), and sepsis (21.2% vs. 11.0, 10.8, 10.0) were more common in deceased patients with COVID-19 patients aged <40 years, although only arrhythmia reaching statistical significance (*p* = 0.039).

**Table 3:** Age-related complications in deceased patients with COVID-19.

**Characteristic**	**Total**	**≤40**	**>40, ≤60**	**>60, ≤80**	**>80**	***p***
*n*	2,492	66	435	1,452	539	
ARDS	1,028 (41.3)	23 (34.8)	160 (36.8)	614 (42.3)	231 (42.9)	0.116
Respiratory failure	1,779 (71.4)	39 (59.1)	288 (66.2)	1,058 (72.9)	394 (73.1)	0.005
Acute myocardial infarction	51(2.0)	0 (0.0)	5 (1.1)	30 (2.1)	16 (3.0)	0.144
Acute myocardial injury	381 (15.3)	5 (7.6)	44 (10.1)	232 (16.0)	100 (18.6)	0.001
Arrhythmia	130 (5.2)	5 (7.6)	11 (2.5)	81 (5.6)	33 (6.1)	0.039
Acute kidney injury	483 (19.4)	5 (7.6)	71 (16.3)	290 (20.0)	117 (21.7)	0.014
Acute liver injury	474 (19.0)	9 (13.6)	87 (20.0)	296 (20.4)	82 (15.2)	0.039
Gastrointestinal bleeding	49 (2.0)	3 (4.5)	10 (2.3)	28 (1.9)	8 (1.5)	0.364
Acute ischemic stroke	38 (1.5)	0 (0.0)	5 (1.1)	17 (1.2)	16 (3.0)	0.018
Sepsis	273 (11.0)	14 (21.2)	48 (11.0)	157 (10.8)	54 (10.0)	0.054
Coagulopathy	560 (22.5)	16 (24.2)	81 (18.6)	335 (23.1)	128 (23.7)	0.201

*COPD, chronic obstructive pulmonary disease; COVID-19, coronavirus disease 2019; Data are reported as number of patients and percentage in the age group (%)*.

### Associations Between Risk Factors and Complications in Deceased Patients With COVID-19

**Figure 3:**
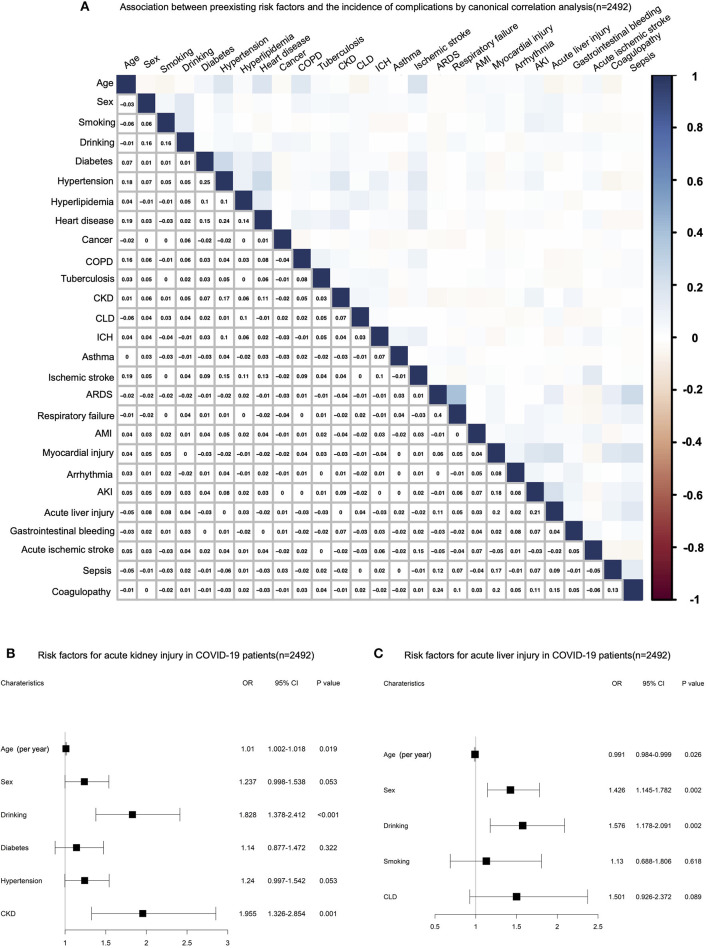
**(A)** Canonical correlation analysis (CCA) for the relationship between preexisting risk factors and the incidence of complications. **(B)** Multivariable logistic regression analysis for risk factors of acute kidney injury (AKI) in deceased patients with COVID-19. **(C)** Multivariable logistic regression analysis for risk factors of acute liver injury in deceased patients with COVID-19.

We further investigated the relationship between preexisting risk factors and the incidence of complications. By canonical correlation analysis, we found a relatively poor correlation between pre-existing risk factors and complications. Higher correlation coefficients (R) were found between male gender (0.075) and alcohol consumption (0.077) with acute liver injury, alcohol consumption (0.092), hypertension (0.077), and history of CKD (0.089) with acute renal failure (Figure 3A). Multivariable logistic regression analysis was used to verify the above correlation. After adjusted for age, sex, and comorbidities, we found younger age (*OR* = 0.991 [95% *CI* 0.984–0.999], *p* = 0.026), male gender (*OR* = 1.426 [95% CI 1.145–1.782], *p* = 0.002), and alcohol consumption (*OR* = 1.576 [95% *CI* 1.178–2.091], *p* = 0.002) were independent risk factors for acute liver injury (Figure 3B), and greater age (*OR* = 1.01 [95% *CI* 1.002–1.018], *p* = 0.019), alcohol consumption (*OR* = 1.828 [95% *CI* 1.378–2.412], *p* < 0.001), and history of chronic kidney (*OR* = 1.955 [95% *CI* 1.326–2.854], *p* = 0.001) were independent risk factors for acute renal failure (Figure 3C).

## Discussions

The COVID-19 pandemic has killed millions of people all over the world, and has frequently overwhelmed healthcare systems in the hardest-hit areas (1). This study provides detailed clinical data on a large sample of patients with COVID-19 in Wuhan, China, the original disease epicenter. As with any infectious disease without specific treatments, host factors are the key determinants of disease severity and prognosis for COVID-19. Identification of risk factors would help to optimize patient management and reduce mortality in future pandemics of the respiratory virus (24).

In this study, we found that greater age, male gender, alcohol consumption, smoking, hypertension, diabetes, cancer, CKD, and ICH were each independent risk factors for death following hospitalization for COVID-19. This finding was in accord with previous large sample studies (7, 25). Among these risk factors, age proved to be the most important, in this study, we get an *OR* of 1.061, but while all other variables are dichotomies, age was treated as a continuous variable, an *OR* of 1.061 means that for each additional year of age, the risk of death increased by 6.1%. The various age-related changes in the geriatric population include deterioration in lung capacity and muscle atrophy, which propagate to physiological impairments, such as reductions of lung reserve, airway clearance, and the defensive barrier function (6). Age-related immune system remodeling, or immunosenescence, is considered to be the major reason for the increased susceptibility of the elderly to viral infection (26–28). Further, preexisting comorbidities are more common in elderly patients. The disparities in the nature of preexisting comorbidities and pathophysiological changes across age groups indicated that patients in different age groups have different risk factors.

With this in mind, we examined differences in clinical manifestations, risk factors, complications, and clinical outcomes among different age groups. Different mortality rates may indicate the possible existence of different risk factors, and the differences in mortality are the main basis for our group setting. Previous studies suggested that children and teenagers (<18) show differences in clinical features and outcomes from adults with COVID-19 (29). In this study, only 473 patients aged <18 were hospitalized due to COVID-19, and they had a less incidence in almost all the symptoms, and only 4 patients died, children seem to have a lower risk of infection than adults and have a lower mortality rate. Similar to pediatric patients, previous studies have shown that people younger than 40 are less likely to become infected with COVID-19, and those infected have a lower mortality rate (14, 26). However, recent national data showed an increase in new infections among young people, an uptick that could not be fully explained by increased testing in these groups (30, 31). The increased infection rate among the young continues to cause mounting deaths in young people. With fewer comorbidities, the cause of death among young people, who presumably possess a stronger immune system, is perplexing. Our analysis revealed cancer and cerebral hemorrhage as the main comorbidities associated with death among patients with COVID-19 younger than 40 years. Due to the small sample size for the patient with cancer and cerebral hemorrhage, though we have more than 6,000 cases in this age group, the *CI* 95% was broad, but very high OR still indicated that cancer and cerebral hemorrhage greatly increased the risk of death. In this group of patients, increasing age appeared to have little effect on the risk of death. This suggests that the reduced daily-life physical abilities due to disability or reduced immunity caused by radiation and chemotherapy for tumors may be the major factors imparting a greater risk of death among young patients with COVID-19.

Previous studies have found that the mortality of patients with COVID-19 begins to increase significantly with age after 40 years old (14), and patients aged 40–80 years were the most influenced by preexisting comorbidities. The risk factors for this age group were similar to those for all patients, probably because they comprised the majority of the entire sample of patients with COVID-19. Patients older than 80 years of age have a particularly high mortality rate (26). In this study, we found that the commonly-reported symptoms of COVID-19, such as fever and cough, had a lesser incidence in the groups older than 80, while falls and compromised mental status on admission were more prevalent. Indeed, advanced age can make the diagnosis more complex, as elderly adults with COVID-19 frequently have atypical manifestations, thus calling for caution in the diagnosis of COVID-19 (5). For people aged >80 years, although there was a higher percentage of patients with multiple comorbidities, older age and male gender were the only factors associated with higher odds of death. Notably, frequent comorbidities, such as hypertension and diabetes were not independently associated with mortality in elderly patients over 80 years of age, as noted in previous studies with smaller sample sizes (32, 33). This result indicates that age-related debilitation and decline in immunity may be the major causes of COVID-19 mortality in the elderly.

Multi-system extrapulmonary complications of COVID-19 had been reported (4, 34), but the differences in the incidence of complications in patients of different age groups have been unknown. In this study, we determined the age-dependence of the prevalence of critical complications in patients dying after hospitalization for COVID-19. We found that coagulopathy, AKI, acute myocardial injury, and acute liver injury were the most common complications occurring after COVID-19 infection. The incidence of most complications increased with age, the new finding was that arrhythmias, gastrointestinal bleeding, and sepsis had a higher incidence in younger patients dying with COVID-19. Disparities by age group in the incidence of extrapulmonary complications provide further evidence that COVID-19 is disproportionally affected by age.

Since complications happened during COVID-19 could be extremely life-threatening, studying the association between previous comorbidities of patients with COVID-19 and present complications may help to take informed measures to reduce mortality, but the association have not been reported. In this study, we found a relatively poor correlation between preexisting risk factors and specific complications. Except for age, sex, and alcohol consumption, we found that only a history of a chronic kidney was independent risk factor for acute renal failure. These results might suggest that extrapulmonary complications are likely related to direct organ damage caused by SARS-CoV-2 and its systemic consequences.

While being the largest cohort study of its type, this study had several limitations. First, it was a retrospective study, thus calling for the identification of primary exposure and outcome, as well as confounding factors, based only upon hospital electronic records from administrative datasets, which may vary in accuracy and reliability among the hospitals. Second, we reviewed complications of deceased in-patients with COVID-19 but were not able to collect corresponding data from patients surviving the disease. Therefore, the incidence of extrapulmonary complications and the association between extrapulmonary complications and the risk of death cannot be explained in this study. Third, we did not study the time from infection to death by methods, such as survival analysis, where age might play a role. Our study demonstrated the effect of age on clinical symptoms, complications, and outcomes, but we did not detail differences among the age groups. In addition, we cannot address the possibility that treatment protocols may have differed among the hospitals based on local experience.

In conclusion, we have described the disproportionally effects of age on the clinical manifestations, risk factors, complications, and outcomes of patients with COVID-19. For the first time, we examined risk factors in patients of different age groups with different mortality. Different from patients aged 40–80 years whose risk factors for death were mainly metabolic comorbidities, cancer and intracerebral hemorrhage were first found to be the main independent risk factors in patients aged <40 years. Age and male gender were the main risk factors for death in patients aged >80 years. We described the incidence of the most common complications of COVID-19 in deceased patients in different age groups, and found that arrhythmias, gastrointestinal bleeding, and sepsis had a higher incidence in younger patients dying with COVID-19. While the association between preexisting risk factors and complications have not been reported, and in this study, we reported a poor correlation. This information gives clinicians a better insight into the effect of age on the clinical manifestations, risk factors, and prognosis of COVID-19, which may guide appropriate remedial measures.

## Data Availability Statement

The raw data supporting the conclusions of this article will be made available by the authors, without undue reservation.

## Author Contributions

HZ, YW, MW, GL, SX, and WW: concept and design. HZ, YW, YH, XLiu, ML, YT, XLi, and GY: acquisition, analysis, or interpretation of data. HZ, YW, MW, and WW: drafting of the manuscript. ML, YT, XLi, and GY: statistical analysis. SX and WW: obtained funding. YH, XLiu, YT, and GL: administrative, technical, or material support. MW and WW: supervision. All authors contributed to the article and approved the submitted version.

## Funding

This study was funded by a COVID-19 Emergency Project from Wuhan Science and Technology Bureau (2020020401010096) and Ministry of Science and Technology of People's Republic of China (2020YFC0841301). The funder was not involved in the study design, collection, analysis, interpretation of data, the writing of this article, or the decision to submit it for publication.

## Conflict of Interest

ML, YT, XLi, and GY are employed by Winning Health Technology Group Co., Ltd. The remaining authors declare that the research was conducted in the absence of any commercial or financial relationships that could be construed as a potential conflict of interest.

## Publisher's Note

All claims expressed in this article are solely those of the authors and do not necessarily represent those of their affiliated organizations, or those of the publisher, the editors and the reviewers. Any product that may be evaluated in this article, or claim that may be made by its manufacturer, is not guaranteed or endorsed by the publisher.

## Abbreviations

COVID-19: the coronavirus disease 2019
ARDS: acute respiratory distress syndrome
AMI: acute myocardial infarction
AIS: acute ischemic stroke
AKI: acute kidney injury
CKD: chronic kidney disease
CLD: chronic liver disease.
