# Supplementary material for: Age-Related Risk Factors and Complications of Patients With COVID-19: A Population-Based Retrospective Study
Source: Front Med (Lausanne). 2022 Jan 11;8:757459. doi: 10.3389/fmed.2021.757459 (PMC8786909; doi:10.3389/fmed.2021.757459)
Supplement: Supplementary file 3 [file Table_3.DOCX]

**Related Computerized Programs With R**

**#** **For comparing of baseline characteristics and outcomes of patients with COVID-19 in different age groups**

data2$age_group<-NA

data2<-within(data2,{

age_group<-NA

age_group[data2$age<=18]<-"0"

age_group[age>18 & age<=40]<-"1"

age_group[age>40 & age<=60]<-"2"

age_group[age>60 & age<=80]<-"3"

age_group[age>80]<-"4"

})

data2$age_group<-as.numeric(data2$age_group)

library(tableone)

myvars<-c('X','XX','XXX', 'Y','YY', 'YYY' …)

catVars<-c('X','XX', 'XXX',…)

nonVars<- c('Y','YY', 'YYY',…)

table<-CreateTableOne(data=data2,vars = myvars, strata="age_group",

factorVars = catVars, addOverall=T)

table1<-print(table, cramVars=catVars, nonnormal=nonVars, exact="M")

**# Univariable and multivariable logistic regression analysis for risk factors of death in the entire cohort of COVID-19 patients.**

Uni<-function(x){

FML<-as.formula(paste0('death~',x))

logistic<-glm(FML,data = data2,family = "binomial")

summary(logistic)

Esum<-summary(logistic)

exp(confint(logistic))

OR<-round(exp(Esum$coefficients[2,1]),3)

CI<-paste0(round(exp(confint(logistic))[2,1:2],3),collapse = "-")

P<-round(Esum$coefficients[2,4],3)

uni<-data.frame('charateristics'=x,

'OR'=OR,

'95 CL'=CI,

'Pvalue'=P)

return(uni)

}

colnames(data2)

Varnames<-colnames(data2)[c(X1,X2…)]

Univar<-lapply(Varnames,Uni)

UNiVar<-ldply(Univar,data.frame)

FML<-as.formula (paste0('death~',paste0(UNiVar[UNiVar$Pvalue<0.1, ]$charateristics,collapse = "+")))

logisticall<-glm(FML,data =data2,family = "binomial")

summary(logisticall)

**#** **Univariable and multivariable logistic regression analysis for risk factors of death in the different age groups of COVID-19 patients.**

data2$age_group<-NA

data2<-within(data2,{

age_group<-NA

age_group[subdata2$age<=40]<-"1"

age_group[age>40 & age<=60]<-"2"

age_group[age>60 & age<=80]<-"3"

age_group[age>80]<-"4"

})

data2$age_group<-as.numeric(data2$age_group)

# subageN N=1,2,3,4

subageN<-data2[data2$age_group==N, ] (N=c(1,2,3,4))

Uni<-function(x){

FML<-as.formula(paste0('death~',x))

logistic<-glm(FML,data = subageN,family = "binomial")

summary(logistic)

Esum<-summary(logistic)

exp(confint(logistic))

OR<-round(exp(Esum$coefficients[2,1]),3)

CI<-paste0(round(exp(confint(logistic))[2,1:2],3),collapse = "-")

P<-round(Esum$coefficients[2,4],3)

uni<-data.frame('charateristics'=x,

'OR'=OR,

'95 CL'=CI,

'Pvalue'=P)

return(uni)

}

colnames(subageN)

Varnames<-colnames(subageN)[c(2:5,7:18)]

Univar<-lapply(Varnames,Uni)

UNiVar<-ldply(Univar,data.frame)

FML<-as.formula (paste0('death~',paste0(UNiVar[UNiVar$Pvalue<0.1, ]$charateristics,collapse = "+")))

logisticall<-glm(FML,data =subageN,family = "binomial")

summary(logisticall)

**# For comparing of compare baseline characteristics, outcomes and age-related complications in deceased COVID-19 patients between different age groups**

deathdata <-data2[data2$death==1, ]

deathdata $age_group<-NA

deathdata <-within(deathdata,{

age_group<-NA

age_group[deathdata $age<=40]<-"1"

age_group[age>40 & age<=60]<-"2"

age_group[age>60 & age<=80]<-"3"

age_group[age>80]<-"4"

})

deathdata $age_group<-as.numeric(deathdata $age_group)

summary(deathdata)

myvars<-c('X','XX','XXX', 'Y','YY', 'YYY' …)

catVars<-c('X','XX', 'XXX',…)

nonVars<- c('Y','YY', 'YYY',…)

table<-CreateTableOne(data= deathdata, strata= "age_group", vars = myvars,

factorVars = catVars)

table1<-print(table, cramVars=catVars, nonnormal=nonVars, exact="M")

**# For canonical correlation analysis**

library(CCA)

jwdata<-deathdata[ ,c(X1,X2…)]

bingfdata<-deathdata[ ,c(Y1,Y2,…)]

X=as.matrix(jwdata)

Y=as.matrix(bingfdata)

correl=matcor(X,Y)

library(corrplot)

corrplot(corr = correl$XYcor, vadd = TRUE, type = "lower", method = "number", diag = FALSE, col = "black", tl.pos = "n",cl.pos="n", tl.cex=0.2,number.cex=0.2)

**# Univariable and multivariable logistic regression analysis for risk factors of the incidence of complications in deceased COVID-19 patients**

Take Arrhythmia as example, other complications were calculated using similar codes:

Uni<-function(x){

FML<-as.formula(paste0('Arrhythmia~',x))

logistic<-glm(FML,data = deathdata,family = "binomial")

summary(logistic)

Esum<-summary(logistic)

exp(confint(logistic))

OR<-round(exp(Esum$coefficients[2,1]),3)

CI<-paste0(round(exp(confint(logistic))[2,1:2],3),collapse = "-")

P<-round(Esum$coefficients[2,4],3)

uni<-data.frame('charateristics'=x,

'OR'=OR,

'95 CL'=CI,

'Pvalue'=P)

return(uni)}

Uni("X")

colnames(deathdata)

Varnames<-colnames(deathdata)[c(14:17,19:29,31)]

Univar<-lapply(Varnames,Uni)

UNiVar<-ldply(Univar,data.frame)

FML<-as.formula (paste0('Arrhythmia~',paste0(UNiVar[UNiVar$Pvalue<0.1, ]$charateristics,collapse = "+")))

logisticall<-glm(FML, data =deathdata,family = "binomial")

summary(logisticall)

**# For drawing of forest maps for the results of multivariable logistic regression analysis**

library(forestplot)

rs_forest <- miltiVar_Acute_kidney_injury

tiff('Figure_Acute_kidney_injury.tiff',height = 5000,width = 6000,res= 600)

tabletext <- cbind(c("Charateristics","\n",rs_forest$Charateristics),

c("OR","\n",rs_forest$OR),

c("95% CI","\n",rs_forest$CI95),

c("P value","\n",rs_forest$Pvalue))

forestplot(labeltext=tabletext,

graph.pos=2,

mean=c(NA,NA,rs_forest$OR),

lower=c(NA,NA,rs_forest$CI95l),

upper=c(NA,NA,rs_forest$CI95h),

)
