# Supplementary material for: Age-Related Risk Factors and Complications of Patients With COVID-19: A Population-Based Retrospective Study
Source: Front Med (Lausanne). 2022 Jan 11;8:757459. doi: 10.3389/fmed.2021.757459 (PMC8786909; doi:10.3389/fmed.2021.757459)
Supplement: Supplementary file 4 [file Data_Sheet_1.PDF]

Supplementary Methods

Standardized collection and processing method

Electronic Medical Record (EMR) from 61 Wuhan hospitals and mobile cabin hospitals were saved as Comma-Separated Values (CSV) file and collected to primary data node. CSV files from each hospital includes the EMR of all COVID-19 patients from admission to discharge.

EMR from primary data node contained both shareable EMR and non-shareable EMR. A standardized processing method was developed to create a functional and semantic interoperability dataset for further analysis. A metadata directory was created to manage all primary EMR. The metadata directory was built up by mapping, cleaning and converting the rule metadata. Since each CSV record type contains its own metadata which is not sharable, a rule-based system map metadata from each CSV file was added to the metadata directory. After data cleaning and data quality validation, incomplete and wrong data was removed, rule metadata including function name parameters, data types were converted to a new form.

Using the metadata directory, a common data model is built up to combine all CSV files into a standardized form. Based on HL7 Clinical Document Architecture and national information classification of medical records, a clinical data information model is mapped with standardized clinical metadata. Then an Observational Medical Outcomes Partnership common data model based COVID-19 data model is generated in this study.

During processing, information includes patients name, identity (ID), address and other personal related information are anonymized. Bias data is detected and cleaned. All data are generated with standardized format. Similar data was removed after similarity detection.

After COVID-19 data model is generated, COVID-19 related clinical information are extracted from data model. Key parameters contain baseline information, end status, physical examination, history, clinical symptoms, laboratory test, treatment related information, Computed Tomography (CT) features, non-drug treatment information.
